# Simultaneous fitting of real-time PCR data with efficiency of amplification modeled as Gaussian function of target fluorescence

**DOI:** 10.1186/1471-2105-9-95

**Published:** 2008-02-12

**Authors:** Anke Batsch, Andrea Noetel, Christian Fork, Anita Urban, Daliborka Lazic, Tina Lucas, Julia Pietsch, Andreas Lazar, Edgar Schömig, Dirk Gründemann

**Affiliations:** 1 Department of Pharmacology, University of Cologne, Gleueler Straße 24, 50931 Cologne, Germany; 2 Center for Molecular Medicine (CMMC), University of Cologne, Joseph-Stelzmann-Straße 52, 50931 Cologne, Germany

## Abstract

**Background:**

In real-time PCR, it is necessary to consider the efficiency of amplification (EA) of amplicons in order to determine initial target levels properly. EAs can be deduced from standard curves, but these involve extra effort and cost and may yield invalid EAs. Alternatively, EA can be extracted from individual fluorescence curves. Unfortunately, this is not reliable enough.

**Results:**

Here we introduce simultaneous non-linear fitting to determine – without standard curves – an optimal common EA for all samples of a group. In order to adjust EA as a function of target fluorescence, and still to describe fluorescence as a function of cycle number, we use an iterative algorithm that increases fluorescence cycle by cycle and thus simulates the PCR process. A Gauss peak function is used to model the decrease of EA with increasing amplicon accumulation. Our approach was validated experimentally with hydrolysis probe or SYBR green detection with dilution series of 5 different targets. It performed distinctly better in terms of accuracy than standard curve, DART-PCR, and LinRegPCR approaches. Based on reliable EAs, it was possible to detect that for some amplicons, extraordinary fluorescence (EA > 2.00) was generated with locked nucleic acid hydrolysis probes, but not with SYBR green.

**Conclusion:**

In comparison to previously reported approaches that are based on the separate analysis of each curve and on modelling EA as a function of cycle number, our approach yields more accurate and precise estimates of relative initial target levels.

## Background

In real-time PCR, fluorescence is recorded at each cycle to monitor the generation of product [1]. Typically, after several cycles with no or minor changes in background fluorescence, there is a short phase with vigorous exponential increase of fluorescence, which then gradually slows down to a plateau phase. In conventional data analysis, for each fluorescence curve a crossing point (Cp) *alias *threshold cycle (Ct) is determined from the visible exponential amplification phase using either the fit point method or the second-derivative method [2]. It is clear that for proper calculation of initial target levels, differences in efficiency of amplification (EA) must be taken into account [3]. Even small EA differences amplify to large apparent differences in mRNA levels [4]. The above methods require the set-up of standard curves from which EA is deduced. The disadvantages of standard curves are (i) the extra effort and cost to set up additional samples *e.g*. by serial dilution, and (ii) non-matching EAs if inhibitors are present and serially diluted [4].

The alternative to using standard curves is to determine EA directly from the samples [5]. The initial exponential amplification can be described in terms of fluorescence (based on the assumption that accumulation of fluorescence is proportional to accumulation of amplification product) by the following equation:

(1) F_x _= F_0_• (EA)^x^

See Table 1 for definition of parameters. Note that in this report, EA has limits of 1 (= no amplification) and 2 (= ideal amplification, *i.e*. complete doubling of target with each cycle); all references to papers where EA runs between 0 and 1 have been transformed by adding 1. Ideally, one would like to determine the individual EA of each sample to determine accurate F_0 _values; F_0 _is directly proportional to the sample target cDNA amount. However, for each trace of fluorescence there are only very few (around 5 to 7) data points with virtually constant EA which can be used for an analysis according to equation 1. In earlier cycles, there is only background fluorescence (*i.e*. amplification product can not be detected for many cycles), and in later cycles the EA declines due to product accumulation [6]. Thus, very few qualified data points combined with considerable measurement error makes direct exponential extrapolation inaccurate. One strategy to improve parameter estimation is to include later points of the fluorescence curve and to adjust EA as a function of cycle number [7-9]. However, we have observed that these approaches can not properly model target fluorescence in detail.

**Table 1:** Definition of parameters of equation 1.

x	Cycle number
F_x_	fluorescence recorded at cycle x
F_0_	virtual initial fluorescence
EA	efficiency of amplification

Very recently, Alvarez *et al*. have introduced into real-time PCR data analysis the useful notion to model the decrease of EA not as a function of cycle number, but as a function of fluorescence, the indicator of product accumulation [10]. This insightful concept is more difficult to apply to data analysis though, since it does not allow direct fitting of flourescence as a simple function of cycle number. Alvarez *et al*. calculate, as F_i+1_/F_i _ratio, amplification efficiencies for each cycle, then fit 2 parameters of a sigmoidal function to these EA values as a function of fluorescence, and finally estimate, with both parameters fixed, F_0 _by iterative discrete fitting. The downsides of this approach are large errors in the F_i+1_/F_i _ratios, non-linear regression with fluorescence as the independent variable (which violates the idea of x having a small or no error), fluorescence data (y axis: F_i+1_/F_i _ratio; x axis: F_i_) on both axes, and fitting twice to the same set of information. Further limitations are indicated in the Discussion.

Based on the innovative concept of modelling EA as a function of amplicon fluorescence, it was our aim here to overcome the defects of the approach of Alvarez *et al*.. As the key improvement, we find that iterative simulation of the PCR process with EA modelled as a Gaussian peak function of amplicon fluorescence yields precise and correct initial EA values, both with hydrolysis probe and SYBR green detection. Our approach includes, for the first time, simultaneous non-linear fitting to determine EA as a common parameter for all samples of a group. Compared to established methods of real-time PCR data analysis, our approach results in more accurate estimates of relative cDNA levels.

## Results

### Modelling EA as a function of target fluorescence

**Figure 1:**
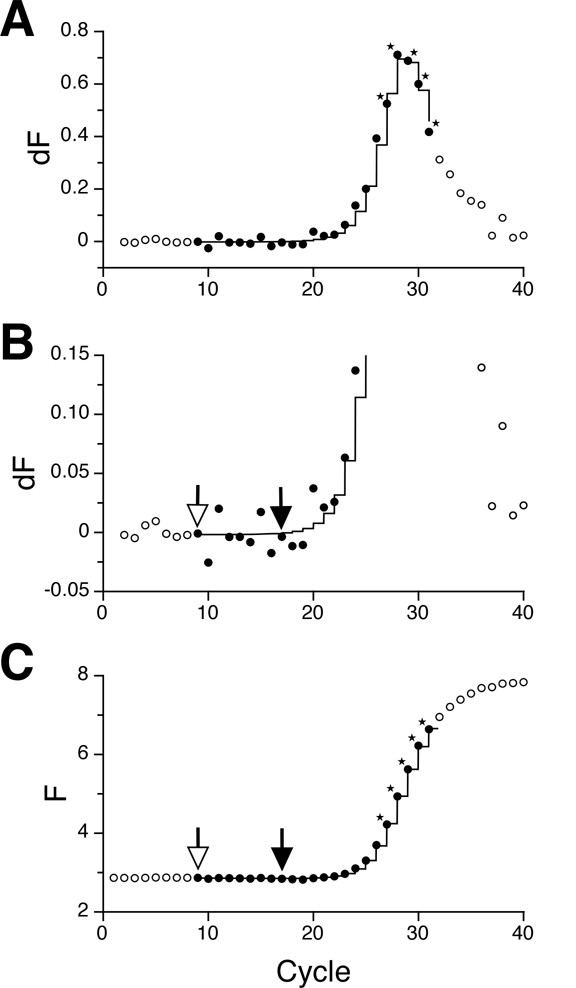
**Selection of points**. (A) Fluorescence difference (dF) as a function of cycle number with data from a hydrolysis probe assay run on a LightCycler™. Filled circles were used for fitting with function EAvPeak; fitting results are displayed as line graph. Circles marked by an asterisk indicate the 5 point peak. (B) As part A, but at higher magnification on the y axis. Points for definition of background fluorescence are selected as follows: each position i, going down cycle by cycle from the 5 point peak, is checked until the dF values of at least 3 points in the interval i-8 to i-1 surpass the reference level, which is the average dF of points i, i+1, and i+2. The upper limit of the background definition interval, denoted by a filled arrow, corresponds to i; the lower limit, denoted by an open arrow, is at i-8. Finally, slope and offset of the background line is determined by linear regression on the raw fluorescence data at i-8 to i. (C) Corresponding raw fluorescence data. Fitting results from function EAv are displayed as line graph. Open circles represent points that were excluded from fitting. Asterisks and arrows as above.

Initially, we tried to fit equation 1 to a limited number (< 6) of data points from the very early visible exponential phase, *i.e*. the first points above background fluorescence. In this phase the EA should still be, as a good approximation, constant and equal to the initial EA. However, this approach was relatively unreliable, even with simultaneous fitting of multiple curves, since there is considerable (random) experimental error (*cf*. background fluorescence differences in Fig. 1B) with every fluorescence reading, yet the last point with the highest fluorescence is always fitted best, even when various weighing options were applied. It is thus necessary to include data points from later cycles in order to mitigate random fluorescence errors. We tested previously reported sigmoid [7,8], logistic [9], and other (*e.g*. asymmetric sigmoid or reverse asymmetric sigmoid) transition functions in order to model target fluorescence as a function of cycle number. All of these, however, showed systematic deviations between calculated and observed fluorescence particularly in the early exponential phase (not shown).

**Figure 2:**
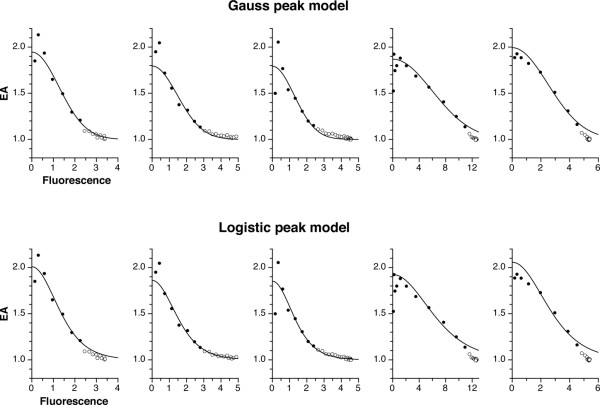
**Efficiency of amplification can be described as a function of fluorescence with a Gauss peak or a logistic peak model**. For each amplicon described in Table 2 (same order, left to right corresponds to top to bottom), the third fluorescence curve (arbitrarily chosen; numbering refers to the Excel raw data file [see Additional file 5]) was analyzed. After subtraction of linear background, EA was calculated as F_i_/F_i-1 _ratio and plotted against fluorescence (F_i_) as shown. To avoid confusion, points with F_i _< 0.1 are not displayed; most of these, because of large errors in EA, lie outside the y axis range. The line graphs were drawn with the Gauss peak function (upper row) or the logistic peak function (lower row). Note that function parameters were not fitted to the points shown, but determined by our stepwise PCR simulation approach based on the raw fluorescence versus cycle number data. Open circles represent data that was not used for fitting.

With the aim of modelling EA as a function of fluorescence [10], we inspected several of our own experimental data sets, plotted as F_i_/F_i-1 _versus F_i _(see Fig. 2). This made us consider a Gauss peak function (y = a * exp [-0.5 * {(x - b)/c}^2^]) and a logistic peak function (y = 4 * a * d/(1 + d)^2 ^with d = exp [-(x - b)/c]) for modelling. Since we expected EA (= F_i_/F_i-1_) to be maximal at F_i _= 0, both functions were simplified by setting b to zero. With the Gauss function, it follows that EA = 1 + (EA_0 _- 1)/exp [F_i_^2^/k] with k = 2 * c^2^. With the logistic function, the analogous equation is EA = 1 + 4 * (EA_0 _- 1) * exp [-F_i_/k]/(1 + exp [-F_i_/k])^2 ^with k = c. Both functions adequately describe F_i_/F_i-1 _as a function of F_i _(Fig. 2). As an important difference, the logistic function always yields higher EA_0 _values than the Gauss function (see below). However, it is not possible to determine which function is more appropriate from this plot, since the critical region of low F_i _is unaccessible, because of very large errors.

In order to describe experimental fluorescence as a function of cycle number, we use an iterative approach that yields all 3 parameters by a single non-linear fitting procedure. Depending on F_0_, the virtual initial target fluorescence, EA_0_, the initial efficiency of amplification, and k, the fluorescence is increased cycle by cycle – with EA adjusted as a function of target fluorescence – up to cycle x. Note that *e.g*. function EA = 1 + (EA_0 _- 1)/exp [F_i_^2^/k] is valid for the plot of F_i_/F_i-1 _versus F_i_. However, in the PCR simulation, it is necessary to calculate – in the other direction – F_i+1 _from F_i_; since EA is not a linear function of F_i_, the available ratio F_i_/F_i-1 _can not be used. Thus, combining EA = F_i+1_/F_i _and EA = 1 + (EA_0 _- 1)/exp [F_i+1_^2^/k] gives F_i _* (EA_0 _- 1)/exp [F_i+1_^2^/k] + F_i _- F_i+1 _= 0. We use the algorithm of Newton [11] to solve this equation by iteration. Note that Alvarez *et al*. have used a F_i+1_/F_i _plot to avoid the need to calculate F_i+1 _from F_i _by Newton iteration.

### Selection of data points

Like previously reported approaches, neither Gauss nor logistic function can reliably model the plateau phase of the PCR fluorescence curve (Fig. 2). We therefore exclude all data points beyond the minimum of the second derivative (approximated by a 5 point peak; see Fig. 1 and Methods for details) from analysis. Also with the fluorescence difference (dF) data, we define the background interval that is modeled by a straight line (Fig. 1B).

### Simultaneous fitting

The EA_0 _values that result from fitting to individual fluorescence curves are still uncertain to an extent that precludes direct use (see below). We thus use simultaneous fitting in the final stage of data analysis to determine an optimal common EA_0_. For this, all associated curves (up to n = 16), with the same points selected as previously for individual fitting, are first pooled into a group by transformation of the cycle numbers (see Methods). Note that the protein of interest and the standard used for normalization, *e.g*. beta-actin, constitute separate groups, since different primers (and probes) are used. Samples with markedly different individual EA_0_s should be gathered into separate groups. With the iterative algorithm described above, a single common EA_0 _is fitted to all curves of a group; at the same time, individual F_0 _and k parameters are fitted for each curve. Based on the shared EA_0_, the final F_0 _values, which are proportional to initial target amount, can be directly used to calculate relative expression levels; for this, normalized ratios, calculated from F_0 _values of the protein of interest and of the corresponding standard protein, are compared.

### Validation

Locked nucleic acid (LNA) hydrolysis probe and SYBR green assays with 5 different targets as shown in Table 2 were performed to resolve whether to use the Gauss function or the logistic function and to validate our approach. For each target, 3 identical dilution series of 5 samples each were processed in parallel; all 15 samples were analyzed as a group. Note that these assays are to some extent imperfect, since they were pipetted and operated by 4 different persons with ordinary skills. In these comparisons, goodness of fit, indicated by chi squared, was not always better with the Gauss function; nevertheless, on average goodness of fit was better *i.e*. chi squared was smaller by a factor of 1.23 (geometric mean; data not shown). Decisively, the Gauss function performed better than the logistic function according to 2 criteria: i) the sum of accuracy errors (error = absolute value of accuracy factor – 1; see Table 2) is smaller, *i.e*. 0.26 (Gauss) vs. 0.44 (logistic). ii) With SYBR green detection of the human GAPDH amplicon, the logistic function yielded a concerted EA_0 _of 2.05; this is significantly higher – the standard deviation from fitting of the concerted EA_0_s was ≤ 0.01 for all 5 targets (data not shown) – than the theoretical upper limit of 2; by contrast, the Gauss function produced an EA of 1.99. Thus, the Gauss function was used for all analyses below.

**Table 2:** Simultaneous analysis of dilution series of 5 targets. For each target, 3 series of 5 samples each were pooled into a single group. Precision is defined as SEM divided by F_0_. The accuracy factor is the geometric mean of the measured dilution steps (calculated from the F_0 _values) divided by the intended dilution step, *i.e*. 4. Raw data is available online as Excel file [see Additional file 5].

Target	Detection	Dilution	EA_0 _group	F_0 _arithmetic mean	SEM	Precision relative error (%)	Accuracy factor
*SLC6A14 *rat	hydrolysis probe	original	1.91	4.1 × 10^-8^	0.06 × 10^-8^	2	1.12 (1.22)^a^
		1 : 4		9.0 × 10^-9^	0.4 × 10^-9^	4	
		1 : 16		1.7 × 10^-9^	0.3 × 10^-9^	17	
		1 : 64		4.6 × 10^-10^	0.2 × 10^-10^	5	
		1 : 256		1.0 × 10^-10^	0.2 × 10^-10^	15	
*SLC22A13 *human	hydrolysis probe	original	1.86	6.1 × 10^-7^	0.2 × 10^-7^	3	0.92 (0.99)
		1 : 4		1.7 × 10^-7^	0.07 × 10^-7^	4	
		1 : 16		4.0 × 10^-8^	0.08 × 10^-8^	2	
		1 : 64		1.2 × 10^-8^	0.05 × 10^-8^	5	
		1 : 256		3.3 × 10^-9^	0.02 × 10^-9^	7	
EMT pig	hydrolysis probe	original	1.79	1.1 × 10^-4^	0.06 × 10^-4^	6	1.02 (1.11)
		1 : 4		3.4 × 10^-5^	0.2 × 10^-5^	6	
		1 : 16		8.4 × 10^-6^	0.4 × 10^-6^	5	
		1 : 64		1.7 × 10^-6^	0.5 × 10^-6^	31	
		1 : 256		4.0 × 10^-7^	0.2 × 10^-7^	6	
ETT chicken	SYBR green	original	1.88	1.1 × 10^-4^	0.2 × 10^-4^	16	0.97(1.03)
		1 : 4		2.3 × 10^-5^	0.5 × 10^-5^	23	
		1 : 16		8.1 × 10^-6^	0.6 × 10^-6^	8	
		1 : 64		1.9 × 10^-6^	0.07 × 10^-6^	4	
		1 : 256		4.9 × 10^-7^	0.2 × 10^-7^	4	
GAPDH human	SYBR green	original	1.99	1.3 × 10^-7^	0.06 × 10^-7^	5	0.99 (1.07)
		1 : 4		4.4 × 10^-8^	0.2 × 10^-8^	6	
		1 : 16		1.1 × 10^-8^	0.03 × 10^-8^	2	
		1 : 64		2.3 × 10^-9^	0.06 × 10^-9^	3	
		1 : 256		5.2 × 10^-10^	0.6 × 10^-10^	11	

^a^: logistic model

Results of Table 2 indicate good precision (median of relative errors: 5%) and accuracy. In order to compare our approach with the standard curve approach [3], each dilution series was analyzed as a separate subgroup as shown in Table 3. With our approach, precision was better by a factor of 1.5 (median of relative errors: 8% vs. 12%); more importantly, accuracy was better by a factor of 2.1 (median of relative errors: 13% vs. 27.5%). Note that our method yields EA_0_s both higher and lower than the corresponding EAs of the standard curve approach. We suppose that this is caused by the LightCycler software for Cp estimation, which can not properly correct a drifting baseline, since the best available baseline adjustment ("arithmetic") simply subtracts a constant offset from all data points. Table 4 shows results from analysis of our data sets with 2 of the tools that are available for data analysis without standard curves. Estimates from LinRegPCR analysis [4] were much less precise (38.5%) and accurate (58.5%). In comparison to the DART-PCR approach [12], which uses the average of individual EAs to calculate F_0 _values, precision was virtually identical (8% vs. 7%); however, accuracy was in favour of our approach by a factor of 1.5 (13% vs. 19.5%). Table 5 suggests that our approach is better than DART-PCR because individual EAs are determined more precisely; SEMs on average (geometric mean) were smaller by a factor of 2.0.

**Table 3:** Comparison of simultaneous analysis and standard curve approach. Data for table 2 was analyzed as 3 subgroups of 5 samples each. In the standard curve approach, the EA was calculated as 10^-1/slope ^with the slope of the regression line. Relative error of accuracy was calculated as the absolute value of 1 – (measured factor/4).

		**Standard curve (Pfaffl) approach**					**Present paper approach**				
			
Target	Dilution step 1 : 4	EA subgroup	Factor arithmetic mean	SEM	Precision relative error (%)	Accuracy relative error (%)	EA_0 _subgroup	Factor arithmetic mean	SEM	Precision relative error (%)	Accuracy relative error (%)
SLC6A14 rat	1	1.77	4.8	0.5	11	21	1.92	4.5	0.2	3	14
	2	1.74	5.0	1.4	28	25	1.92	5.7	1.0	17	42
	3	1.80	3.1	0.9	28	21	1.89	3.6	0.5	14	9
	4		4.7	1.3	28	18		4.9	1.1	23	22
SLC22A13 human	1	2.08	2.9	0.2	7	29	1.86	3.6	0.1	3	10
	2	2.04	5.7	0.2	3	43	1.86	4.3	0.1	3	6
	3	1.82	2.7	0.3	12	33	1.86	3.3	0.2	6	17
	4		6.5	1.4	21	61		3.7	0.4	11	8
EMT pig	1	1.71	3.1	0.1	4	23	1.81	3.3	0.1	2	18
	2	1.76	2.5	0.9	35	38	1.79	4.1	0.3	8	3
	3	1.77	9.6	4.1	42	140	1.78	7.0	3.5	50	75
	4		5.3	1.8	33	33		4.2	1.2	28	5
ETT chicken	1	1.87	6.7	2.0	30	66	1.89	5.3	1.5	27	33
	2	2.01	2.8	0.6	22	31	1.89	2.9	0.7	25	28
	3	1.83	4.9	0.6	12	21	1.86	4.3	0.3	8	7
	4		3.7	0.4	10	7		3.9	0.2	5	3
GAPDH human	1	1.91	2.0	0.1	5	50	2.00	3.0	0.1	3	26
	2	1.92	5.0	0.3	6	26	1.96	4.0	0.3	7	0
	3	1.84	4.9	0.1	1	22	1.99	4.8	0.2	5	20
	4		4.3	0.1	2	6		4.5	0.4	9	12
				*Median:*	12	27.5			*Median:*	8	13

**Table 4:** Analysis with 2 previous approaches that work without standard curves. Data for table 2 was analyzed as 3 subgroups of 5 samples each. For each curve, fluorescence of point 10 was subtracted as background from all points. With the DART-PCR approach, each curve was first analyzed separately for EA. R_0 _values (that correspond to F_0_) were calculated with the average EA for each subgroup. With the LinRegPCR approach, software version 7.2 was used to analyze each curve separately.

		**DART-PCR approach**					**LinRegPCR approach**				
			
Target	Dilution step 1 : 4	mean EA subgroup	Factor arithmetic mean	SEM	Precision relative error (%)	Accuracy relative error (%)	EA range	Factor arithmetic mean	SEM	Precision relative error (%)	Accuracy relative error (%)
SLC6A14 rat	1	1.97	4.7	0.2	5	18	1.79 – 2.04	71.7	57	79	>1000
	2	1.94	6.4	1.6	25	61	1.71 – 2.09	0.94	0.23	24	77
	3	1.86	3.6	0.5	14	10	1.83 – 2.79	>1000	>1000	100	>1000
	4		5.0	1.1	21	26		8.6	8.5	99	114
SLC22A13 human	1	1.70	3.2	0.1	3	21	1.64 – 2.01	6.4	2.9	45	59
	2	1.70	3.3	0.1	2	17	1.68 – 2.24	254	227	89	>1000
	3	1.71	3.0	0.1	1	25	1.52 – 1.93	412	412	100	>1000
	4		3.1	0.1	4	23		36	36	100	797
EMT pig	1		3.0	0.2	6	25	1.67 – 2.02	8.8	7.7	87	121
	2	1.78	3.7	0.2	6	7	1.66 – 1.84	7.2	3.5	49	80
	3	1.87	6.8	2.9	43	69	1.60 – 1.75	11.7	4.9	42	193
	4	1.70	4.2	1.2	29	4		3.4	0.9	25	16
ETT chicken	1	1.87	4.9	1.2	25	22	1.77 – 1.91	3.0	0.6	19	24
	2	1.86	3.0	0.8	26	25	1.76 – 1.88	2.5	0.4	18	37
	3	1.92	4.0	0.3	7	1	1.85 – 1.92	2.4	0.8	32	39
	4		4.0	0.3	7	0		6.4	0.7	12	59
GAPDH human	1	1.86	2.9	0.1	3	28	1.84 – 1.90	4.6	0.4	10	15
	2	1.84	3.4	0.3	8	15	1.78 – 1.87	3.0	0.8	26	25
	3	1.81	4.0	0.2	4	1	1.77 – 1.86	6.3	0.5	7	58
	4		3.8	0.3	7	6		2.8	1.0	35	29
				*Median:*	7	19.5			*Median:*	38.5	58.5

**Table 5:** Comparison of individual EAs: DART-PCR vs. present paper approach. Individual EAs were obtained during data analyses as reported in Tables 3 and 4.

		**DART-PCR approach**				**Present paper approach**				
			
Target	Dilution	EA individual	Arithmetic mean	SEM	Precision relative error (%)	EA_0 _individual	Arithmetic mean	SEM	Precision relative error (%)	***Error ratio***
SLC6A14 rat	original	1.88, 1.91, 1.80	1.92	0.02	1.1	1.83, 1.86, 1.86	1.91	0.01	0.7	***1.59***
	1 : 4	1.96, 2.00, 1.82				1.91, 1.91, 1.84				
	1 : 16	1.96, 2.02, 1.90				1.95, 1.92, 1.91				
	1 : 64	2.03, 1.89, 1.81				1.95, 1.93, 1.96				
	1 : 256	2.03, 1.87, 1.96				1.97, 2.00, 1.90				
SLC22A13 human	original	1.79, 1.85, 1.86	1.70	0.03	1.6	1.94, 1.92, 1.86	1.85	0.02	0.9	***1.76***
	1 : 4	1.78, 1.83, 1.86				1.82, 1.83, 1.89				
	1 : 16	1.66, 1.61, 1.62				1.80, 1.73, 1.88				
	1 : 64	1.63, 1.66, 1.62				1.97, 1.87, 1.81				
	1 : 256	1.65, 1.56, 1.60				1.75, 1.90, 1.82				
EMT pig	original	1.83, 1.83, 1.79	1.78	0.02	1.4	1.84, 1.83, 1.82	1.79	0.01	0.4	***3.14***
	1 : 4	1.85, 1.92, 1.64				1.81, 1.79, 1.76				
	1 : 16	1.68, 1.82, 1.65				1.79, 1.80, 1.78				
	1 : 64	1.75, 1.94, 1.69				1.82, 1.75, 1.80				
	1 : 256	1.77, 1.83, 1.72				1.81, 1.75, 1.75				
ETT chicken	original	1.95, 1.93, 2.06	1.89	0.02	0.9	1.87, 1.89, 1.87	1.88	0.01	0.4	***2.63***
	1 : 4	1.86, 1.81, 1.97				1.89, 1.88, 1.83				
	1 : 16	1.87, 1.81, 1.85				1.87, 1.88, 1.82				
	1 : 64	1.87, 1.92, 1.86				1.92, 1.89, 1.90				
	1 : 256	1.80, 1.84, 1.86				1.88, 1.91, 1.87				
GAPDH human	original	1.86, 1.78, 1.77	1.84	0.01	0.6	2.06, 1.94, 2.02	1.99	0.01	0.5	***1.24***
	1 : 4	1.91, 1.84, 1.82				1.97, 1.99, 2.00				
	1 : 16	1.80, 1.83, 1.79				1.99, 1.98, 1.97				
	1 : 64	1.86, 1.86, 1.85				1.96, 1.94, 2.01				
	1 : 256	1.86, 1.88, 1.83				2.05, 1.97, 1.97				

**Figure 3:**
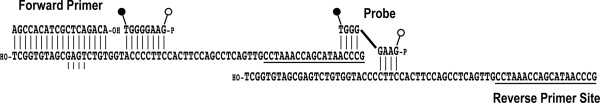
**Proposed mechanism of extraordinary fluorescence growth with LNA hydrolysis probe detection**. The example shown is the GAPDH amplicon (see Materials and Methods) which *e.g*. had an EA_0 _of 2.33 with hydrolysis probe and 2.00 with SYBR green detection. The diagram shows forward primer, LNA hydrolysis probe with 5' fluorophore (filled circle) and 3' quencher (open circle), and the entire amplicon antisense strand, with reverse primer sequence underlined. A 3' phosphate (P) prevents elongation of the probe.

Surprisingly, with some hydrolysis probe assays we obtained EA_0_s definitely higher than 2.00; concurrently, the measured dilution factors of corresponding dilution series were strikingly wrong. With the same primers, but SYBR green instead of hydrolysis probe detection, EA_0_s ≤ 2.00 were determined, and measured matched intended dilution factors. Thus, with LNA hydrolysis probes (Roche Universal Probe Library), efficiency of fluorescence generation can be higher than efficiency of amplification. Extra fluorescence is not caused by the probe alone, since for one amplicon probe #89 gave a higher EA_0 _than the SYBR green assay (2.11 *vs*. 1.86; ≥ 3 samples per group), but for another amplicon detection with same probe matched SYBR green (1.84 *vs*. 1.84). Based on sequence analysis and dedicated experiments we have devised a hypothesis, depicted in Figure 3, to explain additional exponential probe hydrolysis. We suppose that, given matching partial binding sites as indicated, the tightly-binding LNA probe may guide the polymerase to switch to a second antisense strand during synthesis of sense strand. This low-efficiency template-switching [13,14] generates an extended amplicon with two perfect probe binding sites instead of one. The extended amplicon can be extended further by the same mechanism. In support of the model, when CCCA (antisense strand, close to the 5' end; read from right to left) was replaced by GGTG, EA_0 _dropped from 2.27 to 2.08 (3 samples per group). Residual fluorescence growth may be caused analogously by the sequence TGAG (marked by half dashes in the figure) in reverse strand synthesis.

## Discussion

In real-time PCR, without a doubt, it would be optimal to determine an individual EA for each sample. However, it does not seem possible with present experimental technology to determine individual EAs according to equation 1 reliably: very few qualified data points (*i.e*. only the first 5–7 points that rise above background fluorescence with virtually constant EA) combined with considerable measurement error makes direct exponential extrapolation inaccurate. One strategy to improve parameter estimation is to include later points of the fluorescence curve. However, we find that sigmoid [7,8], logistic [9], or other functions can not properly model target fluorescence in detail. Very recently, Alvarez *et al*. have introduced a fundamentally different approach [10]. It appreciates that the decrease of EA is caused by product accumulation [6,15]. This concept allows to embrace even more points for analysis (*i.e*. up to the minimum of the second derivative of fluorescence) than other methods, which use the maximum of the second derivative as an upper limit [9] or the center of selection [12]. Unfortunately, the particular algorithm of Alvarez *et al*., which is based on a sigmoidal function, suffers from a number of disadvantages (see Introduction and below). In the present report we use iterative non-linear fitting with a Gauss function to describe EA as a function of fluorescence. Both approaches use the same number of parameters for fitting, *i.e*. 2 parameters plus the actual result, F_0_. However, our approach has the following advantages over the approach of Alvarez *et al*.: i) Parameters EA_0_, F_0_, and k are fitted directly to the fluorescence *vs*. cycle number data without any data transformation except for inevitable subtraction of background; this avoids additional errors (as in the F_i+1_/F_i _ratios) and preserves error composition. ii) All final parameters are estimated in a single round of fitting. Alvarez *et al*. have rejected direct iterative fitting of F_0 _alongside with their 2 model parameters because of large uncertainty in the estimation of F_0_. Instead, they use an unfavourable algorithm that involves data transformation and fitting twice to the same data set. By contrast, data from Tables 2 and 3 suggests that our Gauss function model allows accurate fitting of the same number of parameters concurrently. iii) EA_0 _can freely surpass 2; this was very instrumental to uncover overestimation of DNA amplification with certain LNA hydrolysis probe assays. By contrast, with the sigmoid function of Alvarez *et al*., EA_0 _is forced to values < 2; to recognize this flaw of formula design, insert a very large T_m_/b ratio in equation 3 of the cited work. iv) Our model is compatible with simultaneous fitting to determine a common EA. Note that simultaneous fitting of EA_0 _is not directly possible with the function of Alvarez *et al*., since there EA_0 _is not a single parameter, but a function of 2 parameters.

In an extensive comparison, the approach of Alvarez *et al*. displayed the lowest quantification error of all methods of individual curve analysis (Fig. 3B and Table 2 in the cited work); similar results were only obtained with EAs estimated from standard curves based on dilution series [3]. We have not applied the approach of Alvarez *et al*. to our data, since, as explained above, the approach is based in parts on unfavorable design. However, our comparison with the widely-used standard curve approach suggests that our approach gives markedly better results (Table 3). Also, we find that our approach is much better in terms of precision and accuracy than the LinRegPCR approach (Table 4). With the DART-PCR approach, which uses the average of individual EAs to calculate F_0 _values, precision was virtually identical; however, accuracy was distinctly better with our approach. We suppose that this is caused predominantly by much more (factor 2.0) precise individual EAs (Table 5). Moreover, with DART-PCR, the mean EAs of 2 amplicons were markedly smaller than the corresponding EA_0_s from our approach; the other 3 were not significantly larger. This is not surprising, since DART-PCR assumes a constant EA which is determined around the second derivative maximum and thereby may underestimate the initial EA.

In spite of these improvements, the F_0 _values that result from fitting to individual fluorescence curves are still uncertain to an extent that precludes direct use (see Table 5, column EA_0 _individual). The individual EAs are useful to identify erratic samples and to judge the quality of primers and probes, but, as was observed previously, they introduce additional error and thus increase data variance [12]. Indeed, in the afore-mentioned comparison of available individual curve analysis methods, accuracy and precision in quantification of experimental dilution series was poor [10]; similarly, with our data sets, the LinRegPCR software yielded the least accurate results (Table 4). Given that determination of F_0 _values from individual EAs is futile because of experimental limitations, then the next best thing is to analyze related samples as a group with a concerted EA. Towards this end, Peirson *et al*. have simply calculated the arithmetic mean of individual EAs [12]. In the present report we introduce simultaneous non-linear regression to determine an optimal EA for all samples of a group. Note that with our large data sets, EA_0 _determined by simultaneous fitting was not dramatically different from the arithmetic mean (compare Arithmetic mean values, Table 5, with EA_0 _group values, Table 2). However, with few samples per group, for example with 6 GAPDH amplicon samples (individual EA_0_s LNA probe: 2.17, 2.25, 2.25; SYBR green: 1.89, 1.96, 1.96), simultaneous fitting (EA_0 _group = 2.01) and arithmetic mean (2.08) may yield markedly disparate results. We suggest that simultaneous fitting provides the best possible EA_0 _that optimally unifies all related fluorescence curves; simultaneous fitting thus contributes to the better performance of our approach. Empirically, for a reliable EA_0 _we would recommend to employ at least 3 samples per group.

Making good use of accurate EA_0_s, our study has revealed that fluorescence generation with some LNA hydrolysis probe assays may overestimate DNA amplification and hence cause incorrect results. To explain this, we assume low-efficiency polymerase template switching that leads to progressive amplicon elongation including additional probe binding sites (Fig. 3). It would thus seem advisable to verify each new LNA hydrolysis probe amplicon with SYBR green detection to avoid spurious fluorescence generation.

## Conclusion

In the present report we introduce a new approach to analyze real-time PCR fluorescence curves without standard curves. Our strategy is based on the useful concept of Alvarez *et al*. to model EA as a function of amplicon fluorescence. As the key improvement, we find that a Gaussian model overcomes the defects of the original sigmoidal model. Iterative simulation of the PCR process up to the minimum of the second derivative of fluorescence yields precise and meaningful initial amplification efficiency values. In the final stage of analysis, a common EA_0 _is fitted simultaneously to all curves of a group of related samples. In comparison to previously reported approaches that are based on the separate analysis of each curve and on modelling EA as a function of cycle number, our approach yields more accurate and precise estimates of relative initial target levels.

## Methods

### Isolation of total RNA and reverse transcription

Total RNA was isolated by the method of Chomczynski and Sacchi [16] from frozen (-80°C) tissues. Reverse transcription was performed as detailed previously [17] with the following modifications: i) RQ1-DNase (Promega, Mannheim, Germany) was used at 1 U/μg total RNA; ii) Random nonamers were used for priming; iii) cDNA synthesis was performed at 42°C.

### PCR

A LightCycler 1.0 apparatus with system 2.0 software (Roche, Mannheim, Germany) was used for real-time PCR. Product accumulation was detected with SYBR green I or with locked nucleic acid hydrolysis probes (TaqMan principle [18]) from the Universal ProbeLibrary (Roche). Primers and probes (see Table 6 for sequences) were selected online with the ProbeFinder (Roche) version 2.35 [19]. A single reaction (total volume 10 μl) contained 1 μl master mix (5 × concentration; LightCycler TaqMan Master; Roche 04735536001), 1 μmol/l each of forward and reverse primer, SYBR Green I at 1 : 30.000 dilution (Invitrogen S7563) or 50 nmol/l probe, and various amounts of cDNA or plasmid DNA. Contamination controls contained water instead of DNA. After enzyme activation (10 min, 95°C), thermocycling consisted of 45 cycles of 10 s at 95°C, 30 s at 55°C, and 1 s at 72°C; velocity of temperature change was 1.1°C/s.

**Table 6:** Sequences of primers and probes.

**Target**	**Forward primer**	**Reverse primer**	**Probe**
*SLC6A14 *rat	CTCAGAGAAGCTGAGGTTTGG	AAGCCACAGAAAGGGAATAAAA	GGATGCTG (#89)
*SLC22A13 *human	GCCCTCAGAGAAGGAAACAG	CTGCTCACAAAGGCCACTC	CTTCCAGC (#11)
EMT pig	CGCTGCCCAACTTTCTCTT	GCTCTATCTCCTTTCTTCCGAGT	CTGGCTGG (#20)
ETT chicken	GCCCCTGTTTGCTTACTTCA	GATCCACCAGAGCGGAAC	GGATGCTG (#89)
GAPDH human	AGCCACATCGCTCAGACA	GCCCAATACGACCAAATCC	TGGGGAAG (#60)

### Analysis of real-time PCR data

Data were analyzed with pro Fit 6.0.6 Software (Quantum Soft, Switzerland) running on a Mac OS X system (Apple, California, U.S.A). Fitting was achieved by non-linear regression with self-written program (SimFitEAv) and function (EAv, EAvPeak, M16EAv) plug-ins. Complete listings (text files) are available online [see Additional file 1] [see Additional file 2] [see Additional file 3] [see Additional file 4]. Functions calculate a single y value from the input; input consists of a single x value and multiple model parameters. Program SimFitEAv analyzes 1 to 16 fluorescence curves simultaneously; it works as follows:

#### Selection of points

First, each fluorescence curve is analyzed separately. The change of fluorescence (dF) as a function of cycle number is used to define an upper limit of useful points and to select points for linear background definition. With the dF data (calculated as fluorescence at cycle i minus fluorescence at cycle i-1), a 5 point peak is identified as the highest sum of dF values of a 5 point sliding window (Fig. 1A). The background fluorescence is modeled individually for each curve by a straight line; this line is defined by a 9 point interval as explained in the legend to Figure 1B. Slope and offset of the background line are determined by linear regression of the corresponding raw fluorescence data. Points preceding the 9 point interval and following the 5 point peak are excluded from further analysis (Fig. 1C). The parameters of a peak function (EAvPeak) are fitted to all remaining points to estimate starting parameters for function EAv. The EAvPeak function basically works like the EAv function described below, but it yields the fluorescence difference between the last and the second-to-last cylce as y output. Note that the number of points used for definition of peak and background were chosen empirically; higher numbers might work as well.

#### Fitting to a single fluorescence curve with variable efficiency of amplification

For all remaining points, linear fluorescence background is subtracted from raw fluorescence. Then, the parameters of function EAv are determined by non-linear regression. In essence, our Gauss model is based on the following equation (exp indicates e raised to the power of its argument in square brackets):

(2) F_i _= F_i-1_• (1 + (EA_0 _- 1)/exp [F_i-1_^2^/k])

See Table 7 for definition of parameters. Note, however, that F_i+1 _is calculated from F_i _by means of Newton iteration [11]. For details, see the function listings provided online as additional files (see above). Amplification is repeated until the cycle number reaches the x input; the final fluorescence is yielded as y output. In other words, each call of function EAv simulates a PCR reaction up to cycle x, starting with F_i _= F_0 _at cycle 1. Apart from linear background, which is added for visual display of final results, this generates a step-wise increase in fluorescence. Individual fitted EA_0 _values are displayed to the user for comparison.

**Table 7:** Definition of parameters of equation 2.

i	current cycle number
F_i_	virtual fluorescence after cycle i
EA_0_	initial efficiency of amplification
k	related to total increase in target fluorescence

#### Simultaneous fitting

In the final stage, a simultaneous fit is made with all curves of a group, with the same points selected as previously for function EAv. Function M16EAv uses a single global EA_0 _parameter for all fluorescence curves; for each curve, parameters F_0 _and k are fitted individually. The same algorithm as in function EAv is used; however, data sets are first joined by transformation of the cycle numbers: curve 1 uses cycle numbers 1 to 50, curve 2 uses 51 to 100 and so forth. Function M16EAv recognizes the input cycle number x and picks the F_0 _and k parameters accordingly. Individual F_0 _values and the common EA_0 _are displayed to the user as the final result.

## Authors' contributions

AB, AN, CF, AU, DL, TL, and JP performed experiments. AL and ES helped to draft the manuscript. DG developed the software, designed experiments, analyzed data, and drafted the manuscript. All authors read and approved the final manuscript.

## Supplementary Material

Additional file 1 Program SimFitEAv. The main program for PCR data analysis to be used as pro Fit (Quantum Soft, Switzerland) plug-in. Click here for file

Additional file 2 Function EAv. Function called from SimFitEAv to be used as pro Fit (Quantum Soft, Switzerland) plug-in. Click here for file

Additional file 3 Function EAvPeak. Function called from SimFitEAv to be used as pro Fit (Quantum Soft, Switzerland) plug-in. Click here for file

Additional file 4 Function M16EAv. Function called from SimFitEAv to be used as pro Fit (Quantum Soft, Switzerland) plug-in. Click here for file

Additional file 5 Original raw data. Excel file with raw fluorescence and cycle data exported from LightCycler. Click here for file

## References

[B1] Bustin SA (2000). Absolute quantification of mRNA using real-time reverse transcription polymerase chain reaction assays. J Mol Endocrinol.

[B2] Rasmussen R, Meuer S, Wittwer C, Nakagawara K (2001). Quantification on the LightCycler. Rapid cycle real-time PCR: methods and applications.

[B3] Pfaffl MW (2001). A new mathematical model for relative quantification in real-time RT-PCR. Nucleic Acids Res.

[B4] Ramakers C, Ruijter JM, Deprez RH, Moorman AF (2003). Assumption-free analysis of quantitative real-time polymerase chain reaction (PCR) data. Neurosci Lett.

[B5] Liu W, Saint DA (2002). A new quantitative method of real time reverse transcription polymerase chain reaction assay based on simulation of polymerase chain reaction kinetics. Anal Biochem.

[B6] Kainz P (2000). The PCR plateau phase – towards an understanding of its limitations. Biochim Biophys Acta.

[B7] Liu W, Saint DA (2002). Validation of a quantitative method for real time PCR kinetics. Biochem Biophys Res Commun.

[B8] Rutledge RG (2004). Sigmoidal curve-fitting redefines quantitative real-time PCR with the prospective of developing automated high-throughput applications. Nucleic Acids Res.

[B9] Tichopad A, Dilger M, Schwarz G, Pfaffl MW (2003). Standardized determination of real-time PCR efficiency from a single reaction set-up. Nucleic Acids Res.

[B10] Alvarez MJ, Vila-Ortiz GJ, Salibe MC, Podhajcer OL, Pitossi FJ (2007). Model based analysis of real-time PCR data from DNA binding dye protocols. BMC Bioinformatics.

[B11] Press WH, Teukolsky SA, Vetterling WT, Flannery BP (1992). Numerical recipes in C: the art of scientific computing.

[B12] Peirson SN, Butler JN, Foster RG (2003). Experimental validation of novel and conventional approaches to quantitative real-time PCR data analysis. Nucleic Acids Res.

[B13] Shammas FV, Heikkila R, Osland A (2001). Fluorescence-based method for measuring and determining the mechanisms of recombination in quantitative PCR. Clin Chim Acta.

[B14] Odelberg SJ, Weiss RB, Hata A, White R (1995). Template-switching during DNA synthesis by Thermus aquaticus DNA polymerase I. Nucleic Acids Res.

[B15] Alvarez MJ, Depino AM, Podhajcer OL, Pitossi FJ (2000). Bias in estimations of DNA content by competitive polymerase chain reaction. Anal Biochem.

[B16] Chomczynski P, Sacchi N (1987). Single-step method of RNA isolation by acid guanidinium thiocyanate-phenol-chloroform extraction. Anal Biochem.

[B17] Gründemann D, Babin-Ebell J, Martel F, Örding N, Schmidt A, Schömig E (1997). Primary structure and functional expression of the apical organic cation transporter from kidney epithelial LLC-PK1 cells. J Biol Chem.

[B18] Heid CA, Stevens J, Livak KJ, Williams PM (1996). Real time quantitative PCR. Genome Res.

[B19] Universal ProbeLibrary Assay Design Center. https://www.roche-applied-science.com/sis/rtpcr/upl/adc.jsp.

